# Milestone Review: The History of Molecular Genetics Analysis of Alzheimer's Disease

**DOI:** 10.1111/jnc.70148

**Published:** 2025-07-02

**Authors:** John Hardy

**Affiliations:** ^1^ UCL Institute of Neurology and Dementia Research Institute London UK

**Keywords:** Alzheimer, amyloid, genetic

## Abstract

Alzheimer research has been driven by genetic findings: from the 1990s until about 2005, by the identification of amyloid precursor protein (*APP*) and presenilin (*PSEN*) mutations, leading to the formulation of the amyloid hypothesis, and then from ~2007 by genome‐wide studies which have led to the increasing appreciation of the importance of microglial insufficiency in the disease pathogenesis. These genome findings have led not only to key mechanistic insights but also to progress in the use of genetic data to predict those at high risk of the disease so that earlier treatment becomes more practical. In this review I will outline these developments and attempts to synthesise the findings into a coherent single view of the disease.

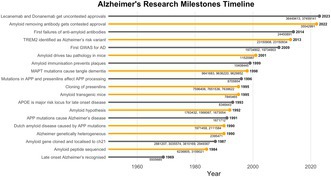

## Graphical abstract

Alzheimer's first case was reported in 1906 (PMID: 8713166) and later expanded upon by Perusini (PMID: 34836480). The chart shows the timeline of the major developments in molecular genetics and animal modelling towards treatments for Alzheimer's disease from 1960 to the present day. The numbers refer to the PubMed IDs (PMIDs) for the relevant papers on the timeline. This review outlines these developments and attempts to synthesise the findings into a coherent single view of the disease.

**Abbreviations**
Aß: amyloid‐beta
APOE: apolipoprotein E
APP: amyloid precursor protein
FTD: fronto temporal dementia
GWAS: genome‐wide association studies
MAPT: microtubule‐associated protein tau
PET: Positron Emission Tomography
PRS: polygenic risk score
PSEN: presenilin

## 1 Early Onset Autosomal Dominant Disease

I have reviewed the identification of the amyloid precursor protein (*APP*) gene before (Hardy 2017). However, it is worth acknowledging that we were led to the amyloid gene (Goate et al. 1991) when we realised that there had to be more than one pathogenic locus for the early onset disease (St George‐Hyslop et al. 1990), causing us to analyze each family we had collected separately, and by the analysis of Dutch Amyloidosis (Van Broeckhoven et al. 1990; Levy et al. 1990), another disease in which amyloid‐beta (Aß) is deposited and which is caused by mutations in the APP gene.

These genetic findings led me (Hardy and Allsop 1991; Hardy and Higgins 1992) and others (Selkoe 1991) to adjudicate on behalf of the amyloid hypothesis of the disease previously proposed by Glenner and Wong (1984) and Masters et al. (1985). The cloning of the microtubule‐associated protein tau (*MAPT*) gene as the locus for the autosomal tangle dementia (Hutton et al. 1998; Poorkaj et al. 1998; Spillantini et al. 1998) allowed the production of mice which deposited tangles (Lewis et al. 2000) and crossing these mice with plaque depositing mice (Holcomb et al. 1998) showed increased tangle formation (Lewis et al. 2001), thus yielding direct experimental proof of the amyloid hypothesis (Hardy et al. 1998).

## 2 Late Onset Disease

The data above constitute clear proof of the amyloid hypothesis in models constructed based on the rare early onset disorder, but there was much argument and discussion about whether it was appropriate to extrapolate from these rare cases to the far more common late onset disease where genetic analysis had not shown either *APP* or presenilin (*PSEN*) mutations. In late onset disease, the only locus known was *APOE*, where E4 was a risk allele (allelic odds ratio ~4) and E2 was protective (allelic odds ratio ~0.4) (Corder et al. 1993). Genetic analysis in late onset disease, first using arrays, yielded an increasing number of risk genes with allelic odds ratios of ~1.1 to ~1.2 (e.g., Lambert et al. 2013). To most researchers' surprise, the majority of these genes were microglial and many were involved (like *APOE*) in lipid metabolism (Jones et al. 2010). Subsequent exome and genome sequencing confirmed this result with the identification of *TREM2* loss of function being the first of these high risk, rare loci to be identified (Guerreiro et al. 2013; Jonsson et al. 2013). In parallel, gene expression analysis in transgenic amyloid depositing mice showed that *TREM2* and several other Alzheimer risk loci were all part of the amyloid response network (Matarin et al. 2015; Salih et al. 2019). The majority of the loci, including *TREM2*, were reduced function variants.

These findings led to a conundrum: early onset disease was caused by increased neuronal production of Aβ whereas late onset disease was predisposed to by reduced function of microglial Aβ responsive genes. Further work showed that many of these genes were upregulated on contact of the microglial with the plaque (Wood et al. 2022).

Based in part on old electron microscopic analyses (Roher et al. 1988; Wisniewski et al. 1989) we have suggested that the resolution of this conundrum is that amyloid deposition is initiated in neuronal membranes, where it disrupts these membranes, which are then engulfed by microglia (Wood et al. 2022) at least in part by an APOE‐dependent mechanism. Within the microglia, the membrane stubs are delipidated (Kaji et al. 2024) and amyloid fibrils are extruded. So we suggest that early onset disease results from overproduction of Aβ and that late onset disease results from a failure in Aβ clearance (Salih et al. 2019).

## 3 Breaking the Pathogenic Cascade Into (At Least) Two Sections

Analysis of genome‐wide association studies of pathology‐confirmed Alzheimer's disease gives large signals at the APOE locus and many low odds ratio associations at microglial loci. In contrast, analysis of PET defined amyloid‐positive signals only shows the APOE signal with dementia in that context depending on the microglial signal. This result breaks the pathogenic cascade into the APOE dependent amyloid deposition and, secondarily to the microglial response (Leonenko et al. 2019; Altmann et al. 2024). A similar conclusion was reached by repeating the amyloid × *MAPT* mice cross when the microglial response was paralysed by *TREM2* knock out. In these *TREM2* knock out mice tangle formation induced by the amyloid deposition was increased (Lee et al. 2021).

These features are all consistent with Alzheimer's pathogenesis having two stages: amyloid deposition dependent on *APOE* genotype and the response to this deposition being largely dependent on the microglial response.

## 4 Predicting Alzheimer's Disease

Besides dissecting pathogenesis, the other major reason for dissecting the genetics of Alzheimer's disease is to identify those at high risk of disease to allow the possibility of early therapeutic intervention. In mendelian disease and in Down's syndrome, prediction is already possible, though the precise age at onset is not certain. In typical disease, in northern European individuals, incorporation of all the GWAS hits with the *APOE* genotypes gives areas under the curve of > 0.75 in pathologically confirmed samples (Escott‐Price et al. 2017). However, the genetic risk and the pathology of disease vary by age, and these variables make individual predictions problematic. The situation in other ethnic groups is less clear because the amount of relevant background data available is insufficient in both Asian and African populations (although East Asian data is becoming available now). This is an area in which progress is needed over the next period.

## 5 GWAS of Intermediate Phenotypes

As the example above showing that amyloid deposition is genetically separable from dementia illustrates, there is more to be gleaned from genetic analysis than only case–control studies. In particular, we have been interested in determining what the genetic basis is of the variability in the rate of disease decline. We have carried out such rate of decline analyses in both progressive supranuclear palsy and Parkinson's disease where the analyses have identified some of the same loci as had been identified by case control analyses, but others which were novel (Jabbari et al. 2021; Tan et al. 2021). In Alzheimer's disease, it is clear that *APOE* is not a progression locus when the analysis is restricted to those who have been diagnosed clinically but with evidence for amyloid pathology either by PET scan or biomarker analysis (consistent with the data summarised above).

Genome‐wide association studies of plasma biomarker analyses also have the potential to yield data on intermediate phenotypes. Work in this area is currently underpowered because of the requirement for both biomarker and genetic data on the same individual as well as a need for consistency in the ages of the individuals who give biomarker samples. Data generated so far confirm the association between *APOE* genotype and amyloid deposition and suggest genetic variability in APP trafficking may influence Aβ production (Stevenson‐Hoare et al. 2023).

One of the most important questions in dementia research now is to understand the molecular bases of co‐pathologies. This is particularly important given the partial success of anti‐amyloid therapies which remove amyloid and reduce tangle pathology but which do not halt clinical decline, presumably because of co‐pathology burden. A recent paper detailing genetic analysis of co‐pathologies, which again is underpowered for practical reasons, nevertheless marks the first progress in this area and shows, for example, that genetic variability at a stroke locus (*COL4A1*) may contribute to vascular pathology and that genetic variability at an FTD locus (*TMEM106B*) may contribute to TDP‐43 pathology (Shade et al. 2024). In other words, this approach, while still underpowered, is already yielding insights into pathogenesis.

## 6 GWAS for Risk Prediction

Ever since the first papers dealing with the use of polygenic risk score to predict Alzheimer risk were published (Escott‐Price et al. 2015), their possible utility in predicting those at high risk and complementing biomarker approaches to risk prediction has been clear. However, their practical use even in white populations has been problematic for two essential reasons: first, as the sample size for GWAS has grown, the diagnostic accuracy used in the test populations, which was never good, has gotten worse (Escott‐Price and Hardy 2022) and the adequate control populations (age matched) have not been used. Thus, prediction accuracy using the latest GWAS data is not better than the earlier and smaller ones. Indeed, it would possibly be better to have two series of GWAS: one for dementia based on the large GWAS with clinical and proxy dementia samples and one based on path‐confirmed samples only, which would, of necessity, be much smaller, and perhaps in which only hits from the dementia GWAS were tested to reduce the Bonferroni correction. Of note, however, we already know that GWAS will never achieve prediction accuracies of > 80% because that is the approximate concordance rate for identical twins (Karlsson et al. 2022).

The above applies to European populations. The situation is much worse in other populations. GWAS in east Asian populations have been initiated and show that the same pathogenic pathways are probably involved, but that the haplotype structures are so different that polygenic risk score (PRS) approaches do not yet allow useful predictions beyond *APOE* (Zhou et al. 2018, 2025). In African populations, work has just begun but has already yielded interesting data suggesting *APOE4* effects are much less in African populations (Rajabli et al. 2022) and also that there is a relatively common internal deletion allele at ABCA7 which is a major risk allele for disease (Cukier et al. 2016). These limited examples show that studying other populations, of course, appropriately benefits them, but that it also benefits all of us as we decipher more fully the mechanisms of risk at all loci.

## 7 The Future… What Do We Need to Do?

The short summary above outlines the remarkable progress we, as a field, have made dissecting the genetics of Alzheimer's disease and, in many ways, the road ahead is clear.

1. Perhaps most importantly, we need to fully investigate the genetics of the dementias in other populations to both find new mechanisms at the loci we already know about and also so we can predict risk more effectively. The collection of neuropathology specimens in these populations and the collection of stem cells from them would be helpful too to dissect variant effects. An example of what can be achieved is the pathogenic Parkinson's *GBA* variant found in Nigeria (Álvarez Jerez et al. 2024).
2. Continuing analysis of neuropathology‐based GWAS, especially in the context of quantitative neuropathologic data.
3. GWAs of rate of decline data based on well phenotyped clinical cohorts may lead to new tractable drug targets.
4. The development of efficient statistical methods for searching for epistasis between loci and the enabling of analytic approaches which can co‐analyse data between jurisdictions without infringing data privacy laws. This would facilitate much of the work where (*n*) are too small in any individual jurisdiction to do definitive studies.

## 8 My Current Views of Pathogenesis of Alzheimer's Disease

In my view, the albeit limited efficacy of anti‐amyloid therapy proves that amyloid hypothesis is, at least partly correct. The continuing failure to define a pathway directly linking amyloid and tau has always puzzled me and my views on this have changed. Genetic evidence, summarised above, implicates amyloid clearance largely by microglia as a key factor in the disease pathogenesis in a late onset disease and more limited data implicates clearance pathways for tau (ubiquitin proteasome) and for synuclein (lysosome) as key to the development of tangle and Lewy body pathologies respectively. Overall, then, these data suggest clearance failure for these highly expressed proteins is key. Of course, clearance pathways are not completely separable and my view now, is that at late age, clearance pathways become overwhelmed (including by environmental damage) and that failure of one clearance system will push other clearance systems into failure rather analogous to over stretched road networks. In such a model, one does not need to hypothesise direct interactions between the deposited proteins: rather they are all victims of age‐related failures in clearance with the precise pathology (and disease) being a function of the which systems are closest to failing. In early onset disease, system failure happens earlier because the proteins are more highly expressed. Such a model obviates the need for elusive ‘toxic species’ and for direct interactions between these proteins. It also allows room for both genetic and environmental predispositions for these prevalent diseases.

## Author Contributions

**John Hardy:** conceptualization, writing – original draft.

## Conflicts of Interest

I have received funding for consulting with Eisai and Eli Lilly.

## Data Availability

The author has nothing to report.
